# Editorial: Role of microbiota in neurocognitive disorders: a developmental origin perspective

**DOI:** 10.3389/fncel.2026.1917819

**Published:** 2026-07-20

**Authors:** Nouha Bouayed Abdelmoula, Hayet Sellami, Sourour Neji, Fabian M. Feiguin, Balkiss Abdelmoula

**Affiliations:** 1 Genomics of Signalopathies at the Service of Precision Medicine LR23ES07 MESRS.tn, Université de Sfax, Faculté de Médecine de Sfax, Sfax, Tunisia; 2 Drosophila UR22ES03, Université de Sfax, Faculté de Médecine de Sfax, Sfax, Tunisia; 3 Laboratory of Fungal and Parasitic Molecular Biology, LR05ES11, Université de Sfax, Faculté de Médecine de Sfax, Sfax, Tunisia; 4 Dipartimento di Scienze della Vita e dell'Ambiente, Università degli Studi di Cagliari, Cagliari, Italy

**Keywords:** developmental origins of health and disease (DOHaD), gut-brain axis, eye-brain axis, microbiota, mycobiota, neurocognitive disorders (NCD), epigenetics, neurodegenerative diseases

The era of brain-centered neurology is giving way to a more integrated biological perspective in which the brain-gut axis and the microbiota redefine neuronal health. In this context, the human mind is now considered as an extension of the microbial ecosystem, shaped by the interaction between the human genome and the microbial metagenome, within the framework of the developmental origins of health and disease (DOHaD).

This research theme entitled “*Role of the microbiota in neurocognitive disorders: a developmental perspective*” brings together 12 review and research articles that collectively map how the microbiota has shaped human evolution and health. The story begins from the earliest stages of life, where the microbiota acts as a key architect shaping the development of the nervous system. Mallick et al. provide in their paper the foundation of this developmental perspective, by examining how early microbial colonization is a prerequisite for neuronal maturation. However, this founding period is fraught with “hidden gateways” to “pathology.” Mohamed et al. offer a cautionary tale about how early iatrogenic disorders-especially NSAID-induced dysbiosis can trigger autoimmune responses and worsen autism spectrum disorders (ASD). This link between microbial metabolic production and neurodevelopment is further hardened by Serrano-Tomás et al., whose meta-analysis identifies p-cresol as a tangible metabolic signature of the severity of ASD, transforming an intestinal by-product into a clinical compass. As the axis matures, the complexity of the actors involved in the brain-gut axis increases. This Research Topic enhances the mycobiome and the molecular master-regulators to the dominant bacteriocentric model. The fungal dimension was treated by Hadrich et al. and Wang et al. These authors provide proof that fungi such as *Candida* and *Saccharomyces*, are not passive residents but active modulators of schizophrenia, depression and attention deficit/hyperactivity disorder (ADHD). Extending this framework, Mohammadi-Pilehdarboni et al. propose an epigenetic bridge mediating long-distance host-microbiome communication, in which non-coding RNAs emerge as central regulators translating microbial signals into epigenetic changes in the brain. Specific neurological functions, including sleep regulation and susceptibility to seizures, are influenced by the intestinal microbiota. Li et al. demonstrated that the ketogenic diet exerts antiepileptic effects in epilepsy by the modulation of the intestinal microbiota, including the enrichment of beneficial taxa such as *Akkermansia* and the depletion of pro-inflammatory or potentially pathogenic microbes such as taxa associated with proteobacteria. Simultaneously, Toraldo et al. propose that dysbiosis can, through the gut-brain axis pathways involving the vagus nerve and the enteric nervous system, contribute to neuroinflammatory processes affecting the brain stem regulation. This may in turn influence the neurotransmitter balance within the main awakening and respiratory control networks, including the preoptic zone (POA) and the ascending reticular activation system (ARAS), potentially leading to impaired levels of neuromodulators such as norepinephrine, dopamine and orexin, as well as relative changes in inhibitory GABAergic signaling. Such a disorder could contribute to reducing the muscle tone of the upper respiratory tract and to altering the respiratory control characteristic of obstructive sleep apnea.

Perhaps the most revolutionary is the work of Kammoun et al., who expand the gut-brain axis concept to the ocular system by proposing a gut-eye axis linking intestinal microbiota to retinal homeostasis and ocular degenerative diseases. Their mini-review highlights emerging evidence associating gut microbiota dysbiosis with conditions such as age-related macular degeneration, glaucoma, and other retinal disorders, suggesting that microbial modulation may influence disease progression and therapeutic response. This work reframe visual neurodegeneration within a systemic host-microbiome interaction network rather than a purely local ocular process.

The final chapter addresses resilience and neuro-degeneration. In the later stages of life, the gut microbiota is increasingly implicated in modulating trajectories of cognitive decline. Singh Solorzano et al. demonstrate, in a memory clinic setting, that microbiota-gut-brain axis mediators such as lipopolysaccharides may serve as differential biomarkers distinguishing Alzheimer's disease (AD) from non-Alzheimer dementias. In parallel, Lin et al. show that the neuropsychiatric burden of Parkinson's disease (PD) is a mirror in the intestine, offering a pathway for personalized neuropsychiatric profiling in elderly patients. They show that the anxiety in PD is associated with distinct gut microbiota signatures compared to healthy individuals, highlighting disease-specific microbial patterns rather than a generalized effect across populations. Their finding suggest that the microbiota-gut-brain axis alterations may differentially shape anxiety-related phenotypes in PD, supporting the potential of microbiome-informed stratification of the patients. Finally, Coretti et al. highlight the aryl hydrocarbon receptor (AHR) pathway as a key molecular interface linking the gut microbiota and the neurodegenerative diseases. Through microbial-derived ligands, particularly those generated via tryptophan catabolism, AHR signaling modulates intestinal and central immune responses, thereby contributing to inflammation and age-associated decline in neurologic function, with implication for both AD and PD.

**Figure 1:**
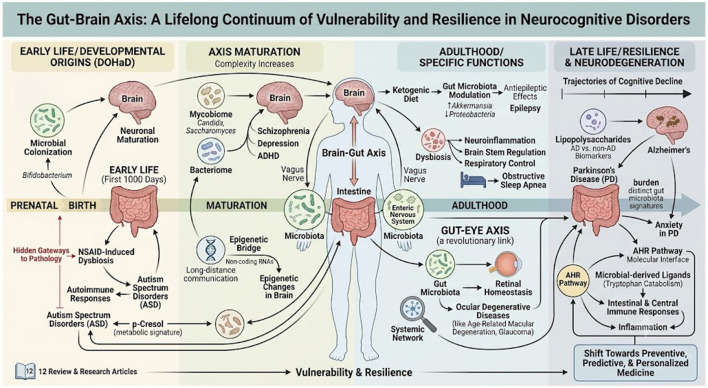
The Gut-Brain Axis: A Lifelong Continuum of Vulnerability and Resilience in Neurocognitive Disorders. This infographic summarizes the 12 articles of the Research Topic by mapping the role of the microbiota across 4 life stages: (1) Early Life/Developmental Origins (DOHaD) — prenatal microbial colonization, NSAID-induced dysbiosis, autism spectrum disorders and p-cresol as metabolic signature; (2) Axis Maturation — mycobiome (Candida, Saccharomyces), epigenetic bridges via non-coding RNAs, schizophrenia, depression and ADHD; (3) Adulthood/Specific Functions — ketogenic diet and epilepsy, dysbiosis, neuroinflammation, obstructive sleep apnea, and the gut-eye axis linking intestinal microbiota to retinal homeostasis; (4) Late Life/Resilience and Neurodegeneration — lipopolysaccharides as Alzheimer's biomarkers, Parkinson's disease gut signatures, anxiety and AHR pathway. The figure concludes with a shift towards preventive, predictive and personalized medicine. Figure generated using artificial intelligence tools.

In conclusion, these 12 scientific papers not only describe an interconnected biological system, but also highlight a lifelong continuum of vulnerability and resilience. By suggesting that neurocognitive disorders across the lifespan, including both early-life neurodevelopmental conditions and late-life neurodegenerative diseases could partly come from microbial programming during the first 1,000 days, this body of work supports a shift toward preventive, predictive and personalized medicine (Figure 1).

Collectively, this Research Topic maps a lifelong continuum of vulnerability and resilience, from early microbial colonization to late-life neurodegeneration, as illustrated in Figure 1.

